# Early speech therapy intervention in children with Autism Spectrum Disorder

**DOI:** 10.1590/2317-1782/e20240245en

**Published:** 2025-08-01

**Authors:** Heloisa Adhmann Ferreira, Paula Mello Pacheco, Thais Helena Ferreira Santos, Daniela Regina Molini-Avejonas

**Affiliations:** 1 Departamento de Fisioterapia, Fonoaudiologia e Terapia Ocupacional, Faculdade de Medicina, Universidade de São Paulo – USP - São Paulo (SP), Brasil.

**Keywords:** Speech and Language Therapy, Autism, Social Communication, Language

## Abstract

**Purpose:**

To analyze the results of speech therapy intervention based on the principles of the DIR/Floortime model in early childhood in children with Autism Spectrum Disorder.

**Methods:**

A longitudinal, quantitative, and prospective manner with direct and indirect intervention, whose target population was children up to three years and eleven months of age, with atypical language development associated with Autism Spectrum Disorder. It led twenty-four speech therapy early intervention sessions based on the DIR Floortime model, in addition to two initial assessment sessions, and two sessions for final assessment.

**Results:**

Twenty children completed the research, with an average age of 29 months at the initial assessment and 36 months in the final assessment. Among the children, 90% already had a diagnosis of Infantile Autism (F84.0). Comparing the results of the Pragmatic Profile, There was an average increase of 0.8 communicative acts and 6.66% in the occupation of the communicative space with statistical significance, as well as a decrease in the use of gestures. There was also a positive glow with moderate significance between “Intentional two-way communication” and the number of acts expressed per minute (the greater the capacity for intentional two-way communication, the greater the number of acts per minute).

**Conclusion:**

When analyzing the pre- and post-intervention results, a consistent and statistically significant evolution is observed. In social communication, skills are interconnected and need to be worked on in a correlational manner, observing the individual needs of each child.

## INTRODUCTION

Autism Spectrum Disorder (ASD) is a neurodevelopmental disorder defined by two main diagnostic criteria. One is the deficits in social communication and interaction with others, and the other is the patterns of restrictive and repetitive behaviors. Both have prolonged duration and are associated with clinically significant impairment^(1)^.

It is possible to identify the first symptoms of autism spectrum disorder in the first year of life, and early diagnosis could be between 12 and 24 months, as confirmed by the DSM-V^(1)^. This early identification is important to refer the child towards interventions due to the specific difficulties of the children with ASD, taking advantage of their development through the neural plasticity of early childhood^(2,3)^.

In this context, scientific evidence highlights the importance of quality in early childhood experiences. These experiences occur in multiple areas of life and are influenced by protective factors, such as cultural, social, and economic encouragement and parental and community support. Early childhood development requires responsible and effective care, including access to health, nutrition, safety, protection, and learning opportunities. Therefore, health professionals and family members must be alert to the signs and characteristics of neurodevelopmental disorders^(3,4)^.

The speech-language pathologists can observe that cognitive-linguistic development undergoes significant transformations during the first years of life^(5)^. Researchers point out that children with autism most often use unconventional and pre-symbolic forms of communication, such as excessive use of gestures and inappropriate vocalizations^(6)^. Thus, the communication of these children ends up being limited to a specific set of communicative intentions, with simple requests and expressions of refusal, which do not establish social interaction and shared attention^(6,7)^.

When aiming for the diagnosis and early intervention of neurodevelopmental disorders, the structural and social aspects of language can be indicators for identifying this delay or deviation^(5)^. In addition to these aspects, pragmatic ability is the main area to be worked on in cases of ASD, considering social cognition and the extralinguistic elements that depend on socialization and interaction, as a very present deficiency in children with this disorder.

Researchers report that pragmatic ability is responsible not only for knowledge of the language structure in which the child is inserted, but also for the rules of coexistence in society and knowledge of the world^(8)^. Thus, pragmatics means the connection between social cognition and the ability to understand extralinguistic elements and make complex inferences about everyday situations.

In this context, the DIR/Floortime model, created by Stanley Greenspan and Serena Wieder in the 1980s, addresses child neurodevelopment and recognizes the importance of early intervention in all areas of the child, prioritizing the development of socioemotional aspects that are known to predict good socialization skills^(9)^.

Greenspan and Wieder^(10)^ address three guiding clinical principles for the model: 1) adapting and promoting interactions with other people, 2) building and cultivating spontaneous interactions, and 3) taking advantage of the child's natural interests as a way of expanding the world. The authors also propose 6 interconnected skills that should be considered in any type of intervention in early childhood: “Self-regulation and interest in the world”, “Formation of relationships, bonds and engagements”, “Intentional two-way communication”, “Behavioral organization, problem-solving and internalization”, “Symbolic and imaginative capacity” and “Emotional, Logical – Abstract Thinking”.

A review study showed that the DIR/Floortime model combined with speech and language pathologist expertise contributed significantly to the development of the pragmatic communication profile in all research participants. This is because there was an increase in interpersonal interactions, social skills, and communicative space, strengthening communicative intentions^(9-14)^.

Within this scenario, this study aimed to analyze the results of speech therapy intervention based on the principles of the DIR/Floortime model in early childhood of children with autism spectrum disorder.

## METHODS

This is a longitudinal, quantitative, and prospective study with therapy interventions. The Ethics Committee of the *Hospital das Clínicas* of the Medicine School at the University of São Paulo approved the research project by opinion 3.633.171/CAEE 15171519.2.0000.0065.

### Population

Children aged from one year to three years and eleven months, whose main complaint from their guardians was atypical language development associated with symptoms of autism spectrum disorder, were included.

Individuals who had more than three absences and/or started speech-language therapy with another professional/methodology during the early intervention process were excluded.

### Material

The Informed Consent Form (ICT), Clinical Anamnesis, Pragmatics - ABFW Child Language Test (2004)^(11)^, and the Functional Emotional Assessment Scale (FEAS)^(9)^ were used.

### Procedure

The Speech-Language Pathology Research Laboratory in Mental Health screening database of the Medicine School of the University of São Paulo was consulted for children who met the inclusion criteria. The children were treated by two different speech-language pathologists from the same laboratory and used the same intervention model based on DIR/Floortime.

The children's guardians were contacted via WhatsApp or phone call to discuss availability and interest in participating in the research. Once everything was set up, direct sessions lasting 45 minutes began.

The first two sessions were dedicated to evaluating the sample. In the first session, a clinical history was taken, and the guardians read and signed the Informed Consent Form. In the second session, the Pragmatics of the Child Language Test (ABFW) and the FEAS were applied.

The Pragmatics protocol is a Brazilian test developed by Dr. Fernanda Miranda Dreux Fernandes. It is considered one of the most comprehensive tests for assessing the pragmatic profile of children with some atypicality in this area. To apply the Pragmatics protocol, a forty-five minutes therapy session was recorded with the children, in which the therapist encouraged them to communicate through problem-solving situations and symbolic games. After filming this session, the five minutes of the most symmetrical interaction were identified, which was the corpus of analysis. This analysis was carried out considering the child's communicative acts, which concern the speaker's ability to initiate a communicative interaction. This is called the communicative space, which indicates the speaker's ability to interact with others and respond assertively and appropriately, and the means of communication used by the child. This is also a valid aspect to be analyzed since children with ASD present non-functional means of communication, differing from the common modes of verbalization, gesticulation, and vocalization in typical pairs^(15)^.

Fernandes et al.^(11)^, through her Pragmatics protocol from ABFW Child Language Test (2004) also describes the socio-communicative skills found in human communication, which can be categorized as more interactive (directed to others) or less interactive (directed to oneself). The most interactive are:

- Object Request - to request a desired object.
- Action Requests - to request that the other act.
- Information Request - to ask the other for information about something.
- Consent Request - to request consent from the other.
- Social Routine Request - to request that the other initiate a routine social interaction game.
- Comment - to direct the other's attention to an object or event.
- Acknowledgement of the Other - to indicate recognition of the other's presence.
- Protest Expression - a manifestation of protest.
- Narrative - to report real or imagined events.
- Shared Game - an organized and shared activity among individuals.
- Exhibition - to attract attention to oneself.
- Exclamatory - to express emotional reaction to an event or situation.
- Protest - to immediately stop an unwanted action.

The less interactive skills are:

- Reactive - to react to some exploration directed at the self.
- Unfocused - communication without focus on the other, object, or environment.
- Self-regulatory - to verbally control one's actions.
- Game - organized but self-centered activity.
- Nomination - to focus attention on an object or event.
- Performative - to perform something familiar.
- Exploratory - to investigate an object, event, or body part.

Regarding the FEAS, the therapist-child session was also filmed for thirty minutes to provide greater detail in the analysis. During this session, the primary caregiver was invited to participate so the child would feel more comfortable. The scores were compared with the FEAS normality reference table, which classifies the child's emotional and functional profile into three categories: deficient, at risk, and normal for his/her age.

This scale enables the observation of the functional and emotional aspects of children who have behavioral and interacting difficulties. The scale is made up of six subtests, which are divided into:

1. Self-regulation and interest in the world, in which the child's interaction with the environment and materials is assessed, as well as the ability to present appropriate reactions to situations during assessments.
2. Formation of relationships, bonds, and engagements. In this subsystem, the child's ability to perceive an adult as a reference and to have a connection with that adult is assessed, as well as to be able to interact with others in an engaged way, sharing attention to something or someone.
3. Intentional two-way communication. It assesses the ability to initiate and close communication circles, that is, to initiate an interaction in an engaged manner and with sustained shared attention, and to close it appropriately. During this subsystem, it is also assessed whether the child uses verbal language in communication circles.
4. Behavioral organization, problem solving, and internalization. It assesses the child's ability to maintain organized play, respecting the limits of others, adhering to previously established rules, and opening opportunities for cognitive flexibility.
5. Representational Capacity, which assesses the ability of symbolic play, so that the child can understand and express abstract concepts through symbols established in the society in which we live.
6. Representational Differentiation. It assesses the child's ability to understand and express abstract concepts, and to infer about the concepts and compare them with each other.

After the assessment, twenty-four direct individual interventions were initiated, lasting forty-five minutes, carried out in person and weekly, based on the principles of DIR/Floortime.

These sessions aimed to expand the child's communication skills, increase verbal means of communication, and improve the socio-emotional skills addressed in the basic intervention model.

All sessions included a toy with a symbolic approach, a toy with a motor approach, and a toy for regulatory use if the child needed it. As a rule, five areas were stimulated: executive functions, communication skills, responsiveness, interaction, and leadership of the child when playing (seeking to achieve symbolic play).

Interventions with the primary caregiver occurred biweekly (indirect intervention), lasting 20 minutes after the child's session. The primary caregiver was invited to participate in half of the session with the child, guided by the therapist, or choose to have a direct conversation with the speech-language therapist responsible for the case, in which communication and basic skills for daily life were addressed.

After the 24 direct and indirect intervention sessions, two sessions were held to reevaluate the children, using the same tests as the initial evaluation.

### Statistical analysis

A descriptive analysis of the characterization data was performed using absolute (n) and relative (%) frequencies. To characterize the population profile, hypothesis tests were performed (chi-square for 3 categories and binomial test for 2 categories), verifying whether the group varied differently between categories.

When comparing the pre- and post-intervention assessments (FEAS and Pragmatics), the Shapiro-Wilk test was applied to verify the normality, followed by the paired t-test. To verify the evolution in the areas of FEAS and Pragmatics, Pearson's correlation was applied, considering the differences between the assessments. The quantitative independent variables with three categories of responses (age, gestational complications, gestational age) used the nonparametric Kruskal-Wallis test in the comparison with FEAS and Pragmatics. The nonparametric Mann-Whitney test was used for the quantitative independent variables with two categories of responses (gender, language delay, and motor delay) compared with FEAS and Pragmatics. The nonparametric Friedman test was used to compare the communicative means, and the comparative analysis of the means before and after the intervention was performed using Student's t-test. A descriptive level of 5% (p<0.05) was assumed for statistical significance. The data were transposed to Excel and analyzed using SPSS version 23 for Windows.

## RESULTS

A total of 20 children completed the research procedure. Among these subjects, 80% were male. The average age of the sample was 29 months at the initial assessment and 36 months at the final assessment. In 90% of cases, children had already been diagnosed with Childhood Autism (F84.0), and the remainder were still in the process of diagnosis.

### Pragmatics test results (ABFW, 2004)

An average increase of 0.8 communicative acts per minute and a p-value <0.001 (<0.05) were observed. The same statistical significance was found in the communicative space, in which there was an average increase of 6.66% of communicative acts per minute, with a standard deviation of 7.78 and a p-value of 0.001 (<0.05). When observing the communicative means, there was a decrease in the use of the gestural means and in some cases, the most used means was replaced by the vocal or verbal means (Table 1)

**Table 1:** Analysis of the difference in pre and post intervention performance according to the ABFW Pragmatics areas

		Mean	SD	Median	Minimum	Maximum	p-value
AC	Pre	3.2	1.1	3.0	1.0	5.4	<0.001
	Post	4.0	1.1	3.9	2.5	6.5	
EC	Pre	36.5	11.6	36.0	24.0	79.0	0.001
	Post	43.2	7.6	42.1	28	65.0	

Paired t-test

**Caption:** AC = number of communicative acts expressed per minute; EC = percentage of occupation of communicative space; SD = standard deviation

### Results in Functional Emotional Assessment Scale (FEAS)

In the “Self-regulation and interest in the world” area of ​​the DIR/Floortime model, an average increase of 1.4 in the overall score and a p-value of 0.001 (<0.05) was noted. In “Formation of relationships, bonds and engagement”, the average increase was 1.8 points and a p-value of 0.001 (<0.05) (Table 2).

**Table 2:** Analysis of the difference in pre and post intervention performance according to FEAS areas

		Mean	SD	Median	Minimum	Maximum	p-value
Subsystem 01	Pre	4.5	1.5	4	1	7	<0.001
	Post	6.1	2.0	6	3	10	
Subsystem 02	Pre	4.1	1.4	4	2	7	<0.001
	Post	5.9	1.9	6	3	11	
Subsystem 03	Pre	3.1	1.3	3	1	5	<0.001
	Post	4.3	1.2	4	2	6	
Subsystem 04	Pre	2.0	1.5	2	0	5	<0.001
	Post	2.9	1.9	3	0	6	
Subsystem 05	Pre	1.6	1.5	1	0	4	<0.001
	Post	2.9	2.3	2	0	7	
Subsystem 06	Pre	0.7	0.6	1	0	2	0.002
	Post	1.4	1.2	1	0	4	

Paired t-test

**Caption:** Subsystem 01 = self-regulation and interest in the world; Subsystem 02 = forming relationships, bonds and engagement; Subsystem 03 = two-way intentional communication; Subsystem 04 = behavioral organization, problem solving and internalization; Subsystem 05 = representational capacity; Subsystem 06 = representational differentiation; AC = number of communicative acts expressed per minute; EC = percentage of occupation of the communicative space

When looking at “Two-way communication”, we noticed an increase of 1.2 with a p-value of 0.001 (<0.05) while in the area “Behavioral organization, problem solving and internalization” there was a smaller improvement, with an average of 0.8, but still with statistical significance and a p-value of 0.001 (<0.05).

In the area “Representational capacity”, it was possible to perceive an average increase of 1.30 and a p-value of 0.001 (<0.05), and for the last area (“Representational differentiation”), an increase of 0.65 and a p-value of 0.002 (<0.05).

Although the subsystems evolved individually and consistently, all children still presented scores within the deficient reference range.

### Statistical correlations

A moderately significant positive correlation was noted between FEAS Subsystem 03 (“Intentional Two-Way Communication”) and the number of acts expressed per minute (the greater the capacity for intentional two-way communication, the greater the number of acts expressed per minute) (Chart 1).

**Chart 1:** Correlation between the evolution observed in FEAS and Pragmatics (ABFW)

	AC		EC	
	*Pearson's ρ*	p-value	*Pearson's ρ*	p-value
Subsystem 01	0.05	0.83	0.20	0.45
Subsystem 02	0.41	0.06	-0.16	0.50
Subsystem 03	0.50	0.04	-0.01	0.95
Subsystem 04	-0.04	0.86	-0.43	0.06
Subsystem 05	0.42	0.06	-0.14	0.56
Subsystem 06	-0.05	0.83	-0.16	0.49

Pearson Correlation

**Caption:** Subsystem 01 = self-regulation and interest in the world; Subsystem 02 = forming relationships, bonds and engagement; Subsystem 03 = two-way intentional communication; Subsystem 04 = behavioral organization, problem solving and internalization; Subsystem 05 = representational capacity; Subsystem 06 = representational differentiation; AC = number of communicative acts expressed per minute; EC = percentage of occupation of the communicative space

There were also two weak correlations that, although not significant, are important to note: 1) “Formation of relationships, bonds and engagements” and communicative acts (positive correlation, the greater the capacity for bonding and engagement, the greater the number of communicative acts per minute); and 2) “Representational capacity” and communicative acts (positive correlation, the greater the representational capacity, the greater the number of communicative acts per minute).

However, there was a weak and negative correlation between Subsystem 04 (Organization of behavior, problem solving, and internalization) and the communicative space, from which it can be inferred that with the increase in behavioral organization, problem solving, and internalization, the children's communicative space decreases.

## DISCUSSION

The critical period for intervention in neurodevelopment occurs before three years old. Many studies indicate that intervention in this age group needs to be direct (therapist-child) and indirect (therapist-caregivers-child) to optimize the process^(16,17)^. Regarding speech-language therapy intervention in early childhood, the intervention should stimulate communication skills, based on playfulness and functionality during the sessions^(18)^.

Pragmatics is the main demand in cases of autism spectrum disorder, the communication with the world. These children have challenges when taking conversational turns, maintaining topics of mutual interest, starting and closing communication circles, and positioning themselves as communicative agents. In this way, communicative acts are translated into clinical reasoning as a means of communication intention. The communicative space occupied translates into a balance between communicative intention and responsiveness, and the means of communication reports a communication functionality in the society in which we live^(19,20)^.

As part of the assessment, the ABFW Pragmatics test allowed observation beyond the structural aspects of language, being important for addressing nonverbal communication, suprasegmental aspects of speech, content and contingency of conversation, communicative skills, reciprocity, and rules of dialogue. In this way, communicative acts are translated into clinical reasoning as a means of communication intention, the occupied communicative space translates into a balance between communicative intention and responsiveness, and the means of communication reports a functionality of communication in the society in which we live^(21)^. It is important to emphasize that nonverbal means should not be understood as “inferior” but as less functional within modern society. Thus, it was possible to perceive that the participants could improve the modus operandi of communication, presenting greater communicative intention, responsiveness, and conversation with the other.

In this context, DIR Floortime provided resources and tools so the speech-language pathology expertise and parent training could occur in the best way, targeting the socio-emotional aspects of development, which are prerequisites for better socialization and learning skills during school. The model also believes that children expand their interests through communication with others, and if communication skills are deficient, their scope for development is also deficient. Increasing the score on the model's reference scale meant increasing interaction skills with others^(22)^.

Self-regulation, a subsystem improved during the research, refers to the ability to guide one's activities, directing them towards long-term goals. This is the primary cognitive and behavioral process that causes an individual to maintain emotional, motivational, and cognitive levels that lead to adaptations, interpersonal relationships, and productive activities^(23)^. Generally, the ability to regulate during interactions between young children is the responsibility of the adults involved, co-regulating the children and supporting them to achieve an emotion, behavior, and cognition balance. However, as children grow and need to be in reciprocal interactions and with intentional communication, this regulation needs to be carried out by the child, referred to as socially shared regulation^(24)^.

Thus, difficulties with self-regulation can result in difficulties with social communication. Approximately 80% of children who suffer from language development disorders have difficulties with self-regulation as a comorbidity^(25)^.

While self-regulation is essential for social interactions, the ability to form bonds and engage in social activities also needs to be active in the child's development, since language occurs through interpersonal interactions in the first thousand days of life. In cases of ASD, children are mostly involved with inanimate objects, reducing the moments of joint activities with their caregivers. This pattern of relationships can influence the development of language and social communication, since the ability to share attention is not developed^(24,25)^.

Thus, noticing statistically significant developments in these self-regulation, formation, and bonding systems is impactful in cases of ASD, especially considering the prerequisite skills for language.

The study was limited in time and subject, but it was able to address the therapeutic process and verify the continuous developments that speech-language therapy brings in early childhood.

## CONCLUSION

When analyzing the pre- and post-intervention results, we observed a consistent and statistically significant improvement in the children's communication and interaction. In social communication, skills are interconnected and need to be worked on in a correlational manner, observing the individual needs of each child and understanding the possibilities that already exist, aiming for functional and abstract communication.
