# Supplementary material for: Early speech therapy intervention in children with Autism Spectrum Disorder
Source: Codas. 2025 Aug 1;37(4):e20240245. doi: 10.1590/2317-1782/e20240245en (PMC12322977; doi:10.1590/2317-1782/e20240245en)
Supplement: Translation

# Translation: Intervenção precoce fonoaudiológica em crianças com Transtorno do Espectro Autista

**DOI:** 10.1590/2317-1782/e20240245pt

**Authors:** Heloisa Adhmann Ferreira, Paula Mello Pacheco, Thais Helena Ferreira Santos, Daniela Regina Molini-Avejonas

**Affiliations:** 1 Departamento de Fisioterapia, Fonoaudiologia e Terapia Ocupacional, Faculdade de Medicina, Universidade de São Paulo – USP - São Paulo (SP), Brasil.

**Keywords:** Fonoaudiologia, Transtorno do Espectro Autista, Comunicação Social, Linguagem

## Abstract

**Objetivo:**

Analisar os resultados da intervenção fonoaudiológica baseada nos princípios do modelo DIR/Floortime na primeiríssima infância em crianças com Transtorno do Espectro Autista.

**Método:**

Estudo longitudinal, quantitativo e prospectivo, com intervenção direta e indireta, cuja população alvo foram crianças com até três anos e onze meses de idade, com atipicidade no desenvolvimento de linguagem relacionado ao Transtorno do Espectro Autista. A pesquisa contou com vinte e quatro sessões de intervenção fonoaudiológica baseando-se nos princípios do DIR/Floortime, somado a duas sessões de avaliação inicial e duas sessões para avaliação final.

**Resultados:**

Vinte crianças concluíram a pesquisa, com idade média de 36 meses na avaliação final. 90% das crianças já tinham diagnóstico de Autismo Infantil (F84.0). Houve aumento médio de 0,8 atos comunicativos e de 6,66% de ocupação do espaço comunicativo com significância estatística, assim como diminuição do uso gestual. Foi observado também uma correlação positiva com significância moderada entre a “Comunicação intencional de duas vias” e o número de atos expressos por minuto (quanto maior a capacidade de comunicação intencional de duas vias, maior o número de atos por minuto).

**Conclusão:**

Ao analisar os resultados pré e pós intervenção, percebeu-se uma evolução consistente e com significância estatística. Na comunicação social, as habilidades são interligadas e precisam ser trabalhadas de modo correlacional, observando as necessidades individuais de cada criança.

## INTRODUÇÃO

O Transtorno do Espectro Autista (TEA) é um transtorno do neurodesenvolvimento definido por dois principais critérios diagnósticos: o déficit na comunicação social e na interação com outras pessoas, e os padrões de comportamentos restritos e repetitivos, ambos de duração prolongada e com comprometimento clinicamente significativo^(1)^.

Sabe-se que é possível identificar os primeiros sintomas do Transtorno do Espectro Autista já no primeiro ano de vida de um indivíduo, sendo possível a realização do diagnóstico precoce, entre 12 e 24 meses, como confirma o DSM-V^(1)^. A identificação precoce é importante pois encaminha a criança para intervenções direcionadas para as dificuldades específicas que as crianças com TEA apresentam, aproveitando as janelas de oportunidades do desenvolvimento por meio da plasticidade neural da primeira infância^(2,3)^.

Neste contexto, existem evidências científicas que destacam a importância da qualidade das experiências na primeira infância. Essas experiências ocorrem em múltiplas áreas da vida e são influenciadas por fatores de proteção, como o incentivo aos aspectos culturais, sociais e econômicos, bem como o apoio dos pais e da comunidade. O desenvolvimento na primeira infância requer cuidados responsáveis ​​e eficazes, incluindo acesso à saúde, nutrição, segurança e proteção, e oportunidades de aprendizagem. Portanto, é fundamental que os profissionais de saúde e familiares estejam atentos aos sinais e características dos transtornos do neurodesenvolvimento^(3,4)^.

Para a fonoaudiologia, é notável que o desenvolvimento cognitivo-linguístico sofre transformações significativas durante os primeiros anos de vida^(5)^. Pesquisadores apontam que crianças com autismo utilizam, na maioria das vezes, formas comunicativas não convencionais e pré-simbólicas, como uso excessivo de gestos e vocalizações inadequadas^(6)^. Dessa forma, a comunicação dessas crianças acaba ficando limitada a um conjunto específico de intenções comunicativas, predominando pedidos simples e expressões de recusa, que não servem para estabelecer interação social e atenção compartilhada^(6,7)^.

Considera-se que ao se objetivar o diagnóstico e a intervenção precoce dos transtornos do neurodesenvolvimento, os aspectos estruturais e sociais da linguagem podem servir como indicadores para identificação desse atraso ou desvio^(5)^. No entanto, para além desses aspectos, a habilidade pragmática ainda é a principal área a ser trabalhada nos casos de TEA, pensando na cognição social e nos elementos extralinguísticos que dependem da socialização e interação com o outro que são tão deficitários em crianças com este transtorno.

Pesquisadores relatam que a competência pragmática é responsável não apenas pelo conhecimento da estrutura da língua em que a criança está inserida, mas também pelas regras de convivência na sociedade e pelo conhecimento do mundo^(8)^. Dessa forma, o domínio da pragmática significa a conexão entre a cognição social e a capacidade de compreender elementos extralinguísticos e de fazer inferências complexas sobre situações cotidianas.

Neste contexto, o modelo DIR/Floortime, desenvolvido por Stanley Greenspan e Serena Wieder na década de 1980, aborda o neurodesenvolvimento infantil e reconhece a importância da intervenção precoce em todas as áreas da criança, priorizando o desenvolvimento de aspectos socioemocionais que é sabidamente preditor de boas habilidades de socialização^(9)^.

Greenspan e Wieder^(10)^ abordam três princípios clínicos norteadores para o modelo, sendo esses: 1) adaptar e promover interações com outras pessoas, 2) construir e cultivar interações espontâneas, e 3) aproveitar os interesses naturais da criança como forma de expandir o mundo. Desse modo, os autores também propõem 6 capacidades socioemocioanais interligadas e que devem ser consideradas em qualquer tipo de intervenção na primeira infância: “Autorregulação e interesse pelo mundo”, “Formação de relacionamentos, vínculos e engajamentos”, “Comunicação bidirecional intencional”, “Organização comportamental, resolução de problemas e internalização”, “Capacidade simbólica e imaginativa” e “Pensamento Emocional, Lógico – Abstrato”.

Quando somado à Fonoaudiologia, sabe-se por estudos de revisão que o modelo DIR/Floortime contribuiu significativamente para o desenvolvimento do perfil pragmático da comunicação em todos os participantes da pesquisa, uma vez que houve aumento das interações interpessoais, das habilidades sociais e do espaço comunicativo, bem como o fortalecimento da intenção comunicativa^(9-14)^.

Dentro deste cenário, o objetivo do presente estudo é analisar os resultados da intervenção fonoaudiológica baseada nos princípios do modelo DIR/Floortime na primeiríssima infância em crianças com Transtorno do Espectro Autista.

## MÉTODO

Estudo longitudinal, quantitativo e prospectivo de intervenção terapêutica. Projeto de pesquisa aprovado pelo Comitê de Ética do do Hospital das Clínicas da Faculdade de Medicina da Universidade de São Paulo, parecer 3.633.171 / CAEE 15171519.2.0000.0065.

### População

Crianças com faixa etária entre um ano e três anos e onze meses, cuja queixa principal dos responsáveis ​​referia-se ao desenvolvimento atípico de linguagem associado aos sintomas do Transtorno do Espectro Autista.

Foram excluídos os sujeitos que ultrapassaram o número de três faltas e/ou que iniciaram atendimento fonoaudiológico com outro profissional/metodologia durante o processo de intervenção precoce.

### Material

Utilizou-se o Termo de Consentimento Livre e Esclarecido (TCLE), Anamnese Clínica, Pragmática - Teste de Linguagem Infantil ABFW (2004)^(11)^ e a Avaliação Funcional Emocional - Functional Emotional Assessment Scale (FEAS)^(9)^.

### Procedimento

Buscou-se no banco de dados de triagem do Laboratório de Pesquisa Fonoaudiológica em Saúde Mental da Faculdade de Medicina da Universidade de São Paulo as crianças que atendiam aos critérios de inclusão. As crianças foram atendidas por duas fonoaudiólogas distintas, ambas pertencentes ao mesmo laboratório, utilizando o mesmo modelo de intervenção baseado no DIR/Floortime.

Os responsáveis ​​pelas crianças foram contatados via WhatsApp ou ligação telefônica para discutir disponibilidade e interesse em participar da pesquisa. Depois de tudo configurado, iniciou-se a realização das sessões diretas com duração de 45 minutos cada.

As duas primeiras sessões foram dedicadas à avaliação da amostra, sendo que na primeira sessão foi realizada uma anamnese clínica e o Termo de Consentimento Livre e Esclarecido foi lido e assinado pelos responsáveis. E na segunda sessão foram aplicados a Pragmática do Teste de Linguagem Infantil (ABFW) e a FEAS.

O protocolo de Pragmática é um teste brasileiro elaborado pela Dra. Fernanda Miranda Dreux Fernandes, e é considerado um dos testes mais completos para avaliar o perfil pragmático das crianças que apresentam atipicidade nessa área. Para aplicação do protocolo Pragmática, foi filmada uma sessão terapeuta-criança de quarenta e cinco minutos, em interação livre com os brinquedos preferidos da criança, na qual a terapeuta instigou a comunicação da criança por meio de situações de resolução de problemas e brincadeiras simbólicas. Após a filmagem, foram identificados os cinco minutos de interação mais simétrica, que serviram para constituir o corpus de análise. Desse modo, a análise foi realizada considerando os atos comunicativos da criança, que diz respeito à capacidade do locutor de iniciar uma interação comunicacional, o espaço comunicativo, que aponta a capacidade do locutor de interagir com o outro e responder de forma assertiva e adequada, e os meios de comunicação utilizados pela criança, que também é um aspecto válido de ser analisado, uma vez que crianças com TEA apresentam meios de comunicação não funcionais, diferindo dos modos comuns aos pares típicos de verbalização, gesticulação e vocalização^(15)^.

Fernandes et al.^(11)^, em seu protocolo de Pragmática do Teste de Linguagem Infantil ABFW (2004) também descreve quais as habilidades sociocomunicativas possíveis de serem encontradas na comunicação humana, que podem ser categorizadas como mais interativas (que se dirige o outro) ou menos interativas (que se dirige a si mesmo). Sendo as mais interativas:

- Pedido de Objeto - para solicitar um objeto desejado.
- Pedidos de Ação - para solicitar que o outro realize uma ação.
- Pedido de Informação - para solicitar ao outro informações sobre algo.
- Pedido de Consentimento - para solicitar consentimento ao outro.
- Pedido de Rotina Social - para solicitar que o outro inicie um jogo de interação social de rotina.
- Comentário - para dirigir a atenção do outro para um objeto ou evento.
- Reconhecimento do Outro - para indicar o reconhecimento da presença do outro.
- Expressão de Protesto - manifestação de protesto.
- Narrativa - para relatar acontecimentos reais ou imaginários.
- Jogo Compartilhado - atividade organizada e compartilhada entre os indivíduos.
- Exibição - para atrair a atenção para si.
- Exclamativa - para expressar reação emocional a um evento ou situação.
- Protesto - para interromper, imediatamente, uma ação indesejada.

E as habilidades menos interativas:

- Reativo - para reagir sobre alguma exploração voltada para o eu.
- Não Focalizada - comunicação sem foco no outro, em objeto ou ambiente.
- Autorregulatório - para controlar verbalmente a própria ação.
- Jogo - atividade organizada, mas autocentrada.
- Nomeação - para focalizar atenção em um objeto ou evento.
- Performativa - para performar algo familiar.
- Exploratória - para investigar um objeto, evento ou parte do corpo.

Em relação ao FEAS, a sessão terapeuta-criança também foi filmada por trinta minutos a fim de proporcionar maior detalhamento na análise, porém durante esta sessão o cuidador principal foi convidado a participar para que a criança se sentisse o mais confortável possível. Os escores foram comparados com a tabela de referência de normalidade do FEAS, cuja o perfil emocional e funcional da criança pode se enquadrar em três categorias, a saber: deficiente, em risco e normal para a idade.

Nesta escala, é possível observar e quantificar os aspectos funcionais e emocionais em crianças que apresentam dificuldades comportamentais e dificuldades de interação. A escala é composta por seis subtestes, que são divididos em:

1. Auto regulação e interesse pelo mundo - no qual é avaliado a interação da criança com o ambiente e os materiais, assim como a capacidade de apresentar reações adequadas para as situações durante as avaliações.
2. Formação de relacionamentos, vínculos e engajamentos - neste subsistema é avaliado a capacidade da criança perceber um adulto de referência e ter uma conexão com esse adulto, assim como de conseguir interagir com o outro de forma engajada, compartilhando atenção sobre algo ou alguém;
3. Comunicação intencional de duas vias - que avalia a capacidade de iniciar e fechar círculos de comunicação, ou seja, de iniciar uma interação de maneira engajada e com sustentação de atenção compartilhada, e fechá-la de maneira adequada. Durante este subsistema também é avaliado se a criança utiliza a linguagem verbal nos círculos de comunicação
4. Organização comportamental, resolução de problemas e internalização - o qual avalia a capacidade da criança de manter uma brincadeira organizada, respeitando os limites do outro, aderindo às regras previamente estabelecidas e abrindo oportunidades para flexibilidade cognitiva.
5. Capacidade Representacional - no qual se avalia a habilidade do brincar simbólico, de modo que criança seja capaz de compreender e expressar conceitos abstratos por meios de símbolos estabelecidos na sociedade em que vivemos.
6. Diferenciação Representacional - avalia a capacidade da criança em, não somente compreender e expressar conceitos abstratos, mas também de inferir sobre os conceitos e compará-los entre si.

Após a avaliação, iniciaram-se vinte e quatro intervenções individuais diretas, com duração de quarenta e cinco minutos, realizadas presencialmente, com periodicidade semanal, baseando-se nos princípios do DIR/Floortime.

Os objetivos das sessões foram ampliar as habilidades comunicativas da criança, aumentar os meios verbais de comunicação e melhorar as capacidades socioemocionais abordadas no modelo base de intervenção.

Todas as sessões tiveram como material um brinquedo com abordagem simbólica, um brinquedo com abordagem motora e um brinquedo para uso regulatório caso a criança necessitasse. E via de regra foram estimulados cinco campos: funções executivas, habilidades comunicativas, capacidade de resposta, interação e liderança da criança na brincadeira (buscando alcançar o brincar simbólico).

As intervenções com o cuidador principal ocorreram quinzenalmente (intervenção indireta), com duração de 20 minutos após a sessão da criança. A cuidadora principal era convidada a participar de metade da sessão com a criança, recebendo orientações da terapeuta, ou podia optar por uma conversa direta com a fonoaudióloga responsável pelo caso, na qual eram abordadas as habilidades comunicativas, habilidades básicas para o dia a dia.

Após a realização das 24 sessões de intervenção direta e indireta, foram realizadas duas sessões para reavaliar as crianças, utilizando os mesmos testes da avaliação inicial.

### Análise estatística

Foi realizada análise descritiva dos dados de caracterização por meio de frequências absolutas (n) e relativas (%). Para caracterizar o perfil populacional foram realizados testes de hipóteses (qui-quadrado para 3 categorias e teste binomial para 2 categorias) a fim de verificar se o grupo variou de forma diferente entre as categorias.

Na comparação entre as avaliações (FEAS e Pragmática) pré e pós intervenção, aplicou-se o teste de Shapiro-Wilk para verificação da normalidade, e em seguida foi aplicado o teste t pareado. Para a verificação da evolução nas áreas do FEAS e da Pragmática foi aplicada a correlação de Pearson considerando as diferenças entre as avaliações. Para as variáveis independentes quantitativas com três categorias de respostas (idade, intercorrência gestacional, idade gestacional) na comparação com o FEAS e a Pragmática foi realizado o teste não paramétrico Kruskal-Wallis. Para as variáveis independentes quantitativas com duas categorias de respostas (sexo, atraso de linguagem e atraso motor) na comparação com o FEAS e a Pragmática foi realizado o teste não paramétrico Mann-Whitney. Na comparação dos meios comunicativos foi utilizado o teste não paramétrico de Friedman e a análise comparativa dos meios pré e pós intervenção foi feita pelo teste t-Student.

Assumiu-se um nível descritivo de 5% (p<0.05) para significância estatística. Os dados foram transpostos para Excel e analisados no programa SPSS versão 23 para Windows.

## RESULTADOS

Um total de 20 crianças completaram o procedimento de pesquisa e, entre os sujeitos, 80% eram do sexo masculino. A idade média da amostra é de 29 meses na avaliação inicial e de 36 meses na avaliação final. 90% das crianças já tinham diagnóstico confirmado de Autismo Infantil (F84.0), e as demais ainda estavam em processo de diagnóstico.

### Resultados do teste de Pragmática (ABFW, 2004)

Em relação aos atos comunicativos, notou-se um aumento médio de 0,8 atos comunicativos por minuto e um valor de p <0,001 (<0,05). A mesma significância estatística foi encontrada em relação ao espaço comunicativo, no qual houve aumento médio de 6,66% de atos comunicativos por minuto, com desvio padrão de 7,78 e valor p de 0,001 (< 0,05). Observando o meio comunicativo foi possível perceber que houve uma diminuição na utilização do meio gestual e em alguns casos o meio mais utilizado foi substituído pelo vocal ou pelo verbal (Tabela 1).

**Tabela 1:** Análise da diferença do desempenho pré e pós intervenção segundo as áreas do Pragmática do ABFW

		Média	DP	Mediana	Mínimo	Máximo	valor de p
AC	Pré	3,2	1,1	3,0	1,0	5,4	<0,001
	Pós	4,0	1,1	3,9	2,5	6,5	
EC	Pré	36,5	11,6	36,0	24,0	79,0	0,001
	Pós	43,2	7,6	42,1	28	65,0	

Teste t pareado

**Legenda:** AC = número de atos comunicativos expressos por minuto; EC = porcentagem de ocupação do espaço comunicativo; DP = desvio padrão

### Resultados Functional Emotional Assessment Scale (FEAS)

Na área “Autorregulação e interesse pelo mundo” do modelo DIR/Floortime, notou-se um aumento médio de 1,4 na pontuação global e um valor p de 0,001 (< 0,05). Em relação à “Formação de relacionamentos, vínculos e engajamento”, o aumento médio foi de 1,8 pontos e valor p de 0,001 (<0,05) (Tabela 2).

**Tabela 2:** Análise da diferença do desempenho pré e pós intervenção segundo as áreas do FEAS

		Média	DP	Mediana	Mínimo	Máximo	valor de p
Subsistema 01	Pré	4,5	1,5	4	1	7	<0,001
	Pós	6,1	2,0	6	3	10	
Subsistema 02	Pré	4,1	1,4	4	2	7	<0,001
	Pós	5,9	1,9	6	3	11	
Subsistema 03	Pré	3,1	1,3	3	1	5	<0,001
	Pós	4,3	1,2	4	2	6	
Subsistema 04	Pré	2,0	1,5	2	0	5	<0,001
	Pós	2,9	1,9	3	0	6	
Subsistema 05	Pré	1,6	1,5	1	0	4	<0,001
	Pós	2,9	2,3	2	0	7	
Subsistema 06	Pré	0,7	0,6	1	0	2	0,002
	Pós	1,4	1,2	1	0	4	

Teste t pareado

**Legenda:** Subsistema 01 = autorregulação e interesse pelo mundo; Subsistema 02 = formando relacionamentos, vínculos e engajamento; Subsistema 03 = comunicação Intencional de duas vias; Subsistema 04 = organização comportamental, resolução de problemas e internalização; Subsistema 05 = capacidade representacional; Subsistema 06 = diferenciação representacional

Ao olhar para “Comunicação de duas vias”, foi possível notar um aumento de 1,2 com valor p de 0,001 (<0,05), enquanto na área “Organização comportamental, resolução de problemas e internalização” notou-se uma melhoria menor, com média de 0,8, mas ainda com significância estatística, com valor p de 0,001 (<0,05).

Em relação à área “Capacidade representacional”, foi possível perceber um aumento médio de 1,30 e um valor p de 0,001 (<0,05), e para a última área (“Diferenciação representacional”), um aumento de 0,65 e um p-valor de 0,002 (< 0,05).

Notou-se que, embora os subsistemas tenham evoluído individualmente de forma consistente, todas as crianças ainda apresentaram pontuações dentro do intervalo de referência deficiente.

### Correlações estatísticas

Notou-se uma correlação positiva moderadamente significativa entre o Subsistema 03 da FEAS (“Comunicação Intencional de Duas Vias”) e o número de atos expressos por minuto (quanto maior a capacidade de comunicação bidirecional intencional, maior o número de atos expressos por minuto) (Quadro 1).

**Quadro 1:** Correlação entre a evolução observada no FEAS e Pragmática (ABFW)

	AC		EC	
	*ρ* de Pearson	Valor de p	*ρ* de Pearson	Valor de p
Subsistema 01	0,05	0,83	0,20	0,45
Subsistema 02	0,41	0,06	-0,16	0,50
Subsistema 03	0,50	0,04	-0,01	0,95
Subsistema 04	-0,04	0,86	-0,43	0,06
Subsistema 05	0,42	0,06	-0,14	0,56
Subsistema 06	-0,05	0,83	-0,16	0,49

Correlação de Pearson

**Legenda:** Subsistema 01 = autorregulação e interesse pelo mundo; Subsistema 02 = formando relacionamentos, vínculos e engajamento; Subsistema 03 = comunicação Intencional de duas vias; Subsistema 04 = organização comportamental, resolução de problemas e internalização; Subsistema 05 = capacidade representacional; Subsistema 06 = diferenciação representacional; AC = número de atos comunicativos expressos por minuto; EC = porcentagem de ocupação do espaço comunicativo

Houve também duas correlações fracas que, apesar de não serem significativas, são importantes de serem observadas: 1) A “Formação de relacionamentos, vínculos e engajamentos” e os atos comunicativos (correlação positiva, quanto maior a capacidade de vínculo e engajamento, maior será a número de atos comunicativos por minuto); e 2) “Capacidade representacional” e atos comunicativos (correlação positiva, quanto maior a capacidade representacional, maior o número de atos comunicativos por minuto).

Contudo, houve uma correlação fraca e negativa entre o Subsistema 04 (Organização de comportamento, resolução de problemas e internalização) e o espaço comunicativo, no qual se infere que com o aumento da organização comportamental, resolução de problemas e internalização, o espaço comunicativo das crianças diminui.

## DISCUSSÃO

Sabe-se que o período crítico para uma intervenção no neurodesenvolvimento se dá antes dos três anos de idade, e muitos estudos indicam que a intervenção nesta faixa etária precisa ser direta (terapeuta-criança) e indireta (terapeuta-cuidadores-criança), para otimizar o processo^(16,17)^. Em relação à intervenção fonoaudiológica na primeira infância, a atuação deve ter como objetivo estimular as habilidades de comunicação, trazendo como base o lúdico e a funcionalidade durante as sessões^(18)^.

Nos casos de Transtorno do Espectro Autista, a principal demanda é a pragmática, a comunicação com o mundo, uma vez que essas crianças apresentam desafios em dar turnos conversacionais, manter temas de interesse mútuo, iniciar e fechar círculos de comunicação e se colocarem como agentes comunicativos. Desta forma, os atos comunicativos são traduzidos em raciocínio clínico como meio de intenção de comunicação, o espaço comunicativo ocupado traduz-se num equilíbrio entre intenção comunicativa e responsividade, e o meio de comunicação reporta uma funcionalidade de comunicação na sociedade em que vivemos^(19,20)^.

Como parte da avaliação, o teste de Pragmática do ABFW proporcionou a observação para além dos aspectos estruturais da linguagem, sendo importante por abordar a comunicação não verbal, aspectos suprassegmentais da fala, conteúdo e contingência da conversação, as habilidades comunicativas, reciprocidade e regras do diálogo. Desse modo, os atos comunicativos são traduzidos para o raciocínio clínico como meio de intenção de comunicação, o espaço ocupado comunicativo traduz-se em um equilíbrio entre intenção comunicativa e responsividade, e o meio de comunicação relata uma funcionalidade da comunicação na sociedade em que vivemos^(21)^. É importante ressaltar que não se deve entender meios não verbais como “inferiores”, mas sim como menos funcionais dentro da sociedade moderna. Assim, foi possível perceber que os participantes conseguiram aprimorar o modus operandi da comunicação, apresentando maior intenção comunicativa, responsividade e conversação com o outro.

Neste contexto, o DIR Floortime forneceu recursos e ferramentas para que, tanto a expertise fonoaudiológica, como a capacitação de pais ocorresse do melhor modo possível visando os aspectos socioemocionais do desenvolvimento, sendo esses pré-requisitos para melhores habilidades de socialização e aprendizagem durante a fase escolar. O modelo também acredita que as crianças expandem seus interesses por meio da comunicação com o outro, e se as habilidades comunicativas estão deficitárias, seu escopo para desenvolvimento também está deficitário, e ao aumentar a pontuação na escala de referência do modelo, significou aumento das habilidades de interação com ao outro^(22)^.

A auto regulação, subsistema aprimorado durante a pesquisa, refere-se à capacidade de orientar as próprias atividades direcionando-as a objetivos de longo prazo. Sendo esse o processo primordial, cognitivo e comportamental, que faz com que um indivíduo mantenha níveis de excitação emocional, motivacional e cognitivo que conduzem a adaptações, relações interpessoais e atividades produtivas^(23)^. O que geralmente ocorre nas interações de crianças pequenas é que a capacidade de regulação durante as interações fica por conta dos adultos envolvidos, co-regulando as crianças e apoiando-as para alcançar o equilíbrio entre emoção, comportamento e cognição. Contudo, à medida que as crianças crescem e necessitam estar em interações recíprocas e com comunicação intencional, o ato de regulação precisa ser realizado pela própria criança, referido como regulação socialmente partilhada^(24)^.

Assim, a dificuldade de autorregulação pode findar em dificuldades de comunicação social. Sabe-se que cerca de 80% das crianças que sofrem com alterações no desenvolvimento da linguagem, apresentam como comorbidade dificuldades no ato de autorregulação^(25)^.

Enquanto a auto regulação é primordial para as interações sociais, a capacidade de formação de vínculos e engajamento social também precisa estar ativa no desenvolvimento da criança, uma vez que a linguagem nos primeiros mil dias acontece por meio de interações interpessoais. Nos casos de TEA, as crianças se envolvem majoritariamente com objetos inanimados, diminuindo os momentos de atividades conjuntas com seus cuidadores, e esse padrão de relacionamento pode influenciar o desenvolvimento de linguagem e comunicação social, uma vez que não se desenvolve a habilidade de atenção compartilhada^(24,25)^.

Assim, perceber evoluções com significância estatística nesses sistemas de auto regulação e formação e vínculo são impactantes nos casos de TEA, pensando principalmente nas habilidades pré-requisitos para a linguagem.

O estudo contou com a limitação de tempo e sujeito, mas foi capaz de abordar o processo terapêutico e verificar as evoluções contínuas que a terapia fonoaudiológica traz na primeiríssima infância.

## CONCLUSÃO

Ao analisar os resultados pré e pós-intervenção, observou-se uma melhora consistente e estatisticamente significativa na comunicação e interação das crianças. Na comunicação social, as habilidades são interligadas e precisam ser trabalhadas de modo correlacional, observando as necessidades individuais de cada criança e compreendendo as possibilidades já existentes, ambicionando a comunicação funcional e abstrata.
